# Can therapeutic drug monitoring increase the safety of Imatinib in GIST patients?

**DOI:** 10.1002/cam4.1286

**Published:** 2018-01-07

**Authors:** Wei Zhuang, Jing‐Dun Xie, Shan Zhou, Zhi‐Wei Zhou, Yi Zhou, Xiao‐Wei Sun, Xiu‐Hong Yuan, Min Huang, Si Liu, Shuang Xin, Qi‐Biao Su, Hai‐Bo Qiu, Xue‐Ding Wang

**Affiliations:** ^1^ Institute of Clinical Pharmacology School of Pharmaceutical Sciences Sun Yat‐Sen University Guangzhou 510006 China; ^2^ State Key Laboratory of Oncology in South China Collaborative Innovation Center for Cancer Medicine Sun Yat‐Sen University Cancer Center Guangzhou 510060 China; ^3^ The first affiliated hospital Sun Yat‐Sen University Guangzhou 510080 China; ^4^ College of health science Guangdong Pharmaceutical University Guangzhou Guangzhou 510006 China

**Keywords:** Adverse effects, GIST, Imatinib, N‐demethyl‐Imatinib, pharmacokinetics

## Abstract

Imatinib at 400 mg daily is the standard treatment for patients affected with CML and GIST. The intervariability in plasma concentration is very significant. In many reports, a good therapeutic effect is attributed to an adequate concentration of Imatinib. However, few studies have been conducted to investigate the association between plasma concentration and side effects. Besides, no upper concentration limit of Imatinib plasma concentration detection has been established. The correlation of Imatinib trough concentrations (C_min_) with adverse effects (AEs) was described here. Plasma samples were obtained from patients after 3 months treatment with Imatinib (steady state, *n* = 122). Liquid chromatography/ tandem mass spectrometry was used to determine the concentration of Imatinib and its metabolite NDI. The incidence of myelosuppression was increased significantly with the increased Imatinib trough plasma concentration. The plasma level of Imatinib and NDI in patients who developed myelosuppression are 1698.3 ± 598.6 ng/mL and 242.1 ng/mL, respectively, which were significantly higher than those in patients who did not (1327.2 ± 623.4 ng/mL,* P *=* *1.75 × 10^‐4^; 206.3 ng/mL,* P *=* *0.006). Estimated exposure thresholds of Imatinib and NDI were 1451.6 ng/mL with ROC_AUC_ (95%CI) of 0.693 (0.597–0.789) and 207.1 ng/mL with ROC_AUC_ (95%CI) of 0.646 (0.546–0.745), respectively. Multivariate regression confirmed the correlation of Imatinib C_min_ with myelosuppression. Other side effects such as fluid retention and rash were not found to be correlated with Imatinib concentrations. These results suggest that trough concentration of Imatinib should be taken into consideration to increase the safety of Imatinib therapy in GIST patients.

## Introduction

Imatinib mesylate (IM, formerly known as CGP 57148B), the first approved selective tyrosine kinase inhibitor (TKI), is currently approved as standard care in patients with BCR‐ABL‐positive chronic myeloid leukemia (CML) and gastrointestinal stromal tumors (GIST) with impressive 6‐ and 8‐year survival rates of 88–95% 1, 2. However, IM is suspended in one in 2–4 patients because of the intolerance or unsatisfactory efficacy 3. Interpatient differences in therapeutic response may be partially due to pharmacokinetic (PK) variability. PK variability, which is mainly caused by genetic, demographic, and environmental factors, is manifested by a broad range of trough plasma levels (C_min_) in patients on the same dosage 4, 5, 6.

In the recent decade, based on the relationship of concentration with response reported in several retrospective studies, researchers proposed that a good clinical response could be attributed to an adequate plasma concentration of IM. On the basis of those findings, Therapeutic Drug Monitoring (TDM) was recommended in IM therapy to monitor the concentration higher than 1000 ng/mL and 1100 ng/mL for CML and GIST, respectively 7, 8.

Since IM is usually administered for a prolonged period, rational management of its side effects is of great importance. Identifying and managing toxicity is therefore key to ensure long‐term therapeutic benefits. The main side‐effects of IM, such as myelosuppression and periocular edema, have been observed in nearly 50% of patients. Nausea, diarrhea, hypophosphatemia, musculoskeletal symptoms, rash, fatigue, and headache are other common side‐effects that have been reported in 15–40% of patients 9. Although most of these side effects were classified as grade 1–2 (non‐life‐threatening) according to Common Terminology Criteria for Adverse Events, the quality of life could be adversely affected to a significant degree by physical and psycho‐social discomfort 10. Although many clinical researches have been conducted to investigate the association between plasma concentration and therapeutic effects, few studies have investigated the association between IM plasma concentration and its toxicity in GIST.

IM is metabolized predominantly by CYP3A4 to its major circulating active metabolite (CGP74588) 11, 12, which is an N‐desmethyl metabolite (NDI). The potency of NDI in vitro is similar to that of IM. Therefore, even if its real impact remains unclear, NDI concentration should be determined in TDM.

Herein we investigated the clinical significance of IM PK at steady state, and the correlation between IM and its main metabolite NDI exposure and the occurrence of specific adverse events. Furthermore, we provide the threshold concentration of the concentration‐related side effects in GIST.

## Materials and Methods

### Patient recruitment

From 2014 to 2016, a total of 122 GIST patients in Sun Yat‐Sen University Cancer Center in Guangzhou, China were enrolled in this study. Exclusion criteria were uncontrolled systemic disease, poor compliance and receiving CYP3A4 or CYP3A5 inhibitor such as St John's Wort, cimetidine. Inclusion criteria were ≥18 year‐olds with adequate hematological, renal and hepatic functions, histologically or molecular‐diagnosis‐confirmed GIST and Eastern Cooperative Oncology Group performance status (ECOG PS) ≤2. Written informed consent was obtained from all participating subjects. This study was approved by the ethical committee of Sun Yat‐Sen University Cancer Center. This trial was registered at ClinicalTrials.gov with number NCT03092128.

All patients were treated with IM at 400 mg daily for at least 3 months. All adverse events were documented and graded according to NCI Common Terminology Criteria for Adverse Events v4.03. Toxicity assessment: physical examination and routine laboratory tests (hematology and biochemistry assessments) were performed once a month by investigators. Myelosuppression grading was based on neutrophil (NEU) and white blood cell (WBC) count. NEU or WBC counts mildly hypocellular or ≤25% reduction from normal cellularity for age is defined as grade 1 myelosuppression; Grade 2 is moderately hypocellular or >25 ‐ <50% reduction from normal cellularity for age; Grade 3 is severely hypocellular or >50 ‐≤75% reduction cellularity from normal for age; Grade 4 is aplastic persistent for longer than 2 weeks.

### Quantification of trough level concentration

Blood samples were collected 24 h (22–26 h) after last dosage (steady‐state trough level, *n* = 122). Blood samples (3 mL each) were collected into EDTA polypropylene tubes, centrifuged at 1000*g* for 10 min for plasma separation. The remaining samples were used for germline mutation detection. All the blood samples were frozen in −80°C refrigerator until analysis. Liquid chromatography and tandem mass spectrometry (LC/MS‐MS) was used to determine the plasma concentrations of IM and NDI 13. Deuterated N‐demethyl‐Imatinib was used as the internal standard. The method was linear over 0.01–10 *μ*g/mL for Imatinib and N‐demethyl‐Imatinib (NDI). For both analyzed compounds, the relative standard deviation (RSD) of intra‐day precision ranged from 2.6% to 8.1% and RSD of the inter‐day precision was in the range of 3.2%–7.3%. The intra‐day errors were between −3.7% and 8.1%, whereas the inter‐day errors were between −3.4% and 7.9%. All the detailed and thorough validation based on FDA guidelines was performed to indicate that this method is sensitive, stable and specific for our pharmacokinetic and pharmacogenomics study in which GIST patients were treated with Imatinib.

### Data analysis and statistical methods

To correlate with side effects, IM plasma trough levels at steady state were grouped into four categories based on distribution according to four quartiles, as summarized in Table 1. Chi‐square test was used to determine the influence of the four PK categories across various adverse reactions groups. The best exposure threshold to predict adverse effects and its performance was evaluated with Receiver operating characteristics (ROC) analyses (in terms of area under the ROC‐curve; ROC_AUC_ and sensitivity/specificity). Concentrations of IM and NDI (C_min_) at steady state (*n* = 122) were correlated with factors including age, gender, body weight, localization, mutation states, surgery, and body surface area (BSA) using logistic regression analysis, these factors were stepwise included in a logistic regression model and retained if the model was significantly improved. *P* < 0.05 was considered statistically significant. All statistical tests were performed using SPSS 21.0.

**Table 1:** Clinical characteristics of patients enrolled. (*n* = 122)

Characteristics	No. of Patients
Median age, years (median, [range])	55, [44‐63]
Gender (Male/Female)	65/57
Median BMI, (median, [range])	20.95, [19.50‐23.98]
Median BSA, m^2^ (median, [range])	1.72, [1.61‐1.81]
Surgery, n (Surgery/non‐surgery)	78/44
Localization, n (Stomach/Intestines/Others)	90/14/18
Mutation, n (*KIT*/*PDGFRA*/Wild Type/Unreported)	65/0/19/38

Data are presented as M (median) with P25‐P75 (Percentile: 25%‐75%), mean ± standard deviation or amount.

## Results

### Demographic characteristics and trough plasma levels of IM and its metabolite NDI

**Figure 1:**
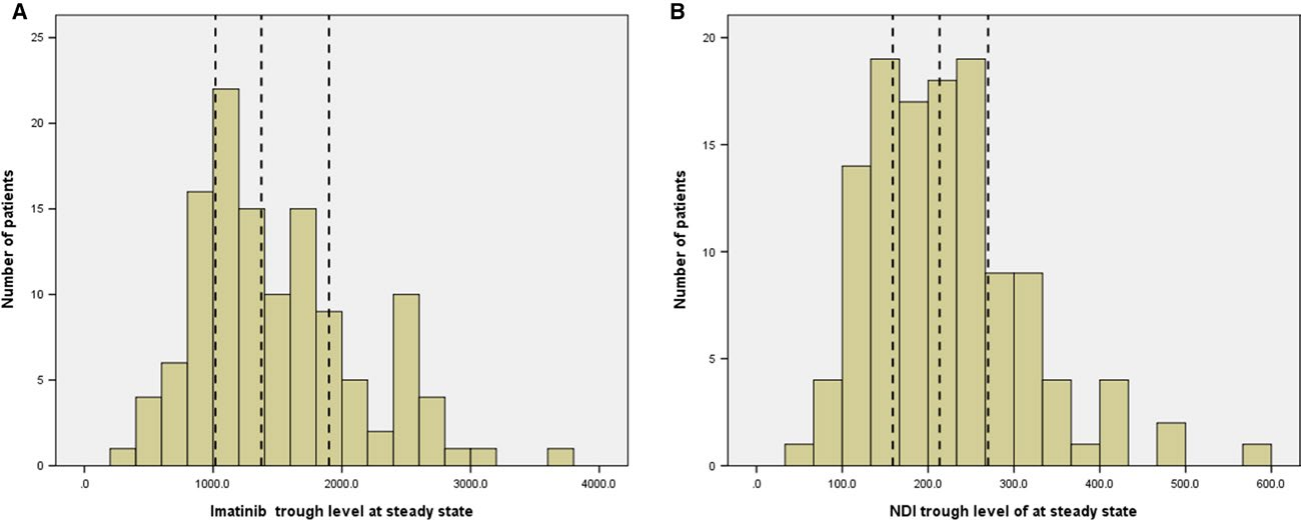
The distribution of the steady‐state trough plasma concentrations of Imatinib (A) and NDI (B) at 400 mg daily (*n* = 122). The vertical dashed lines indicate quartiles. (NDI:* N*‐desmethyl Imatinib)

Table 1 shows the clinical characteristics of patients. There were 122 patients, 65 men and 57 women, with a median age of 55 years (range, 18–70 years). The mean BSA was 1.72 m^2^ (range, 1.71–1.82 m^2^). The mean (± SD; median, [range]) trough concentrations of IM and NDI were 1506.7 (±636.8 ng/mL; 1377.7 ng/mL, [397.0–3614.9 ng/mL]) and 222.1 (±91.5 ng/mL; 214.0 ng/mL, [65.3–575.6 ng/mL]), respectively (*n* = 122). Figure 1 shows the distributions of IM and NDI trough levels at steady state. And the concentration ratio of metabolite to parent drug at steady state was 0.159 ± 0.061 overall, but appeared to be slightly higher at lower IM concentrations: 0.198, 0.161, 0.152, and 0.125 for Q1, Q2, Q3, and Q4 quartiles, respectively.

### Correlation of the IM and NDI trough levels with adverse‐effects rates

**Figure 2:**
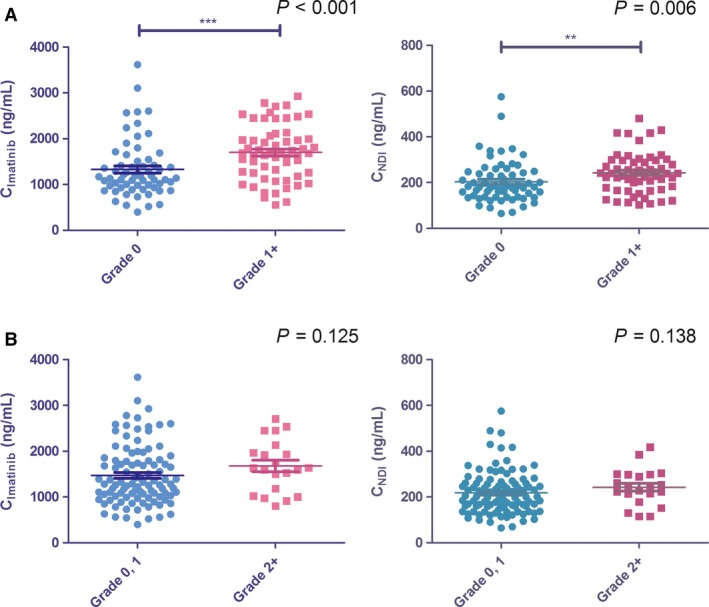
Imatinib (A) and NDI (B) trough concentrations in GIST patients with and without myelosuppression.

Frequencies of major AEs for all grades (0, 1, and 2^+^) observed in the first 3 months are summarized in Table 2. There are no significant difference in the types and grades of AEs among patients in all four PK categories, except for myelosuppression, which occurred more frequently in patients in Q4 than those in Q1. Furthermore, the concentration dependency of IM‐induced myelosuppression was not only in the categorizing exposure levels (Grade 0 vs. 1^+^,*χ* ^2^=17.552, *P *=* *5 × 10^‐4^), but also over the whole range of trough concentrations (Grade 0 vs. 1^+^,*P *=* *2 × 10^‐4^) (Fig. 2). Trough plasma levels of NDI showed a similar pattern, that is, it was just correlated with myelosuppression (Grade 0 vs. 1^+^, *χ* ^2^=11.864, *P *=* *0.006), not with other AEs.

**Table 2:** Association analyses between adverse effects and categorizing exposure levels

Quartiles	*n*	Myelosuppression (Grade)				Edema Limbs (Grade)				Rash (Grade)				Myalgia (Grade)			Periocular Edema (Grade)			Conjunctival Hemorrhage (Grade)			
		0	1	2^+^	*P* a	0	1	2^+^	*P* a	0	1	2^+^	*P* a	0	1^+^	*P* a	0	1^+^	*P* a	0	1	2^+^	*P* a
Imatinib																							
Q1	30	21	5	4	<0.001	25	2	3	0.674	27	3	0	0.560	22	8	0.891	13	17	0.266	28	2	0	0.779
Q2	31	22	7	2	0.125	25	3	3	0.762	24	5	2	0.244	25	6		7	24		27	4	0	0.870
Q3	31	11	13	7		23	5	3		25	6	0		24	7		8	23		26	4	1	
Q4	30	9	13	8		26	3	1		26	4	0		22	8		7	23		27	2	1	
NDI																							
Q1	32	20	8	4	0.006	27	1	2	0.073	26	4	0	0.852	20	10	0.331	12	18	0.387	26	4	0	0.907
Q2	31	23	6	2	0.138	26	4	1	0.363	25	4	2	0.244	24	7		7	24		28	3	0	0.870
Q3	37	12	17	8		20	6	5		25	6	0		23	8		7	24		28	2	1	
Q4	35	12	16	7		26	2	2		26	4	0		26	4		9	21		26	3	1	

a *P* value of row 1 was grade 0 versus 1 + ; *P* value of row 2 was grade 0&1 versus 2+.

### Univariate concentration‐adverse effect analysis

**Figure 3:**
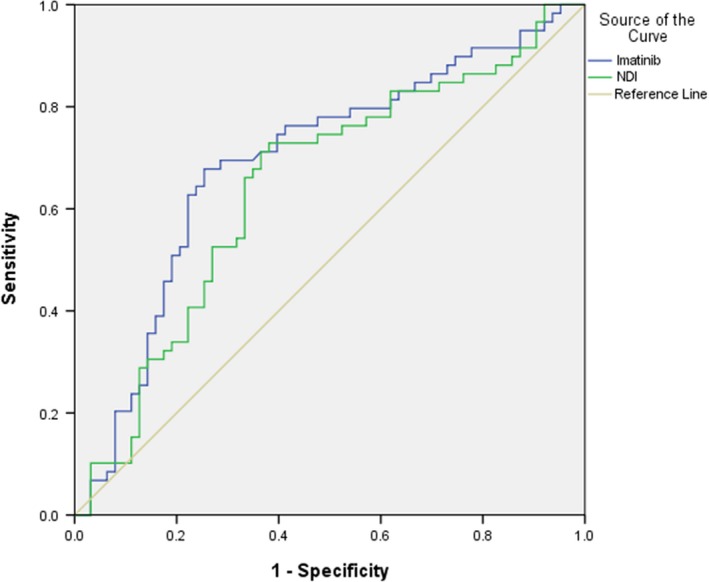
Performance of IM and NDI concentration thresholds to predict myelosuppression with receiver operating characteristic analyses.

Significant correlations with IM and NDI concentrations were found only for myelosuppression, but not for other side effects, including edema limbs, rash, myalgia, periocular edema, and conjunctival hemorrhage (Table 2). Trough concentrations of IM and NDI were significantly correlated with the occurrence of myelosuppression (Figure 3). For IM, threshold (sensitivity/specificity) was 1451.6 ng/mL (72.2% /75.0%) for the presence of myelosuppression, with the range of ROC_AUC_ of 0.597–0.789; and for NDI, threshold (sensitivity/specificity) was 207.1 ng/mL (70.4%/61.8%) with the range of ROC_AUC_ of 0.546–0.745 (Table 3).

**Table 3:** Best exposure threshold of Imatinib and NDI to predict myelosuppression and its performance

	Threshold value [C_min_(400 mg)]	*P*	Area Under the Curve (95% Cl)
Imatinib	1451.6 ng/mL	1.75 × 10^‐4^	0.693 (0.597‐0.789)
NDI	207.1 ng/mL	0.006	0.646 (0.546‐0.745)

### Multivariate analysis of IM plasma concentrations with its side effects taking into account potential confounding factors

When taking into account potential confounding factors including demographic‐ and disease characteristics into a stepwise logistic regression analysis, IM quartiles level was found to be predictive of development of myelosuppression, with the odds of myelosuppression increasing 1.928‐fold per quartiles increase in C_min_. The NDI level was not survived in the multivariate analysis (Table 4).

**Table 4:** Multivariate logistic regression of Imatinib trough levels for the prediction of myelosuppression

	Parameter	*P*	OR, (95%CI)
Myelosuppression (Grade 0 vs. 1^+^)	C_Imatinib_	2.91 × 10^‐4^	1.928, (1.352‐2.751)
	Constant	0.001	0.179

## Discussion

As TKIs are often administered for a prolonged period, rational management of TKI‐associated adverse effects is of great importance. Adverse effects (AEs) can lead to poor adherence to therapy, and it is well known that severe [Common Terminology Criteria for Adverse Events (CTCAE) grades 3‐4] toxicities or chronic CTCAE grade 2 AEs may demand either a change in, or interruption in, treatment. For IM, the archetypal TKI, notable increase in adverse effects was reported in this decade 9, 14, 15. This study for the first time reported that in GIST patients IM‐induced myelosuppression is IM and NDI plasma concentration dependent, with the upper limit of 1451.6 ng/mL with ROC_AUC_ (95% CI) of 0.693 (0.597–0.789) for IM, and the upper limit of 207.1 ng/mL with ROC_AUC_ (95% CI) of 0.646 (0.546–0.745) for NDI. Other AEs, such as edema limbs, rash, myalgia, periocular edema, and conjunctival hemorrhage side effects were not found to be concentration‐dependent, and TDM cannot decrease the risk of the occurrence of these AEs. Furthermore, both IM and its major metabolite are correlated with myelosuppression, indicating that detection of both compounds is very important. Up to date, this is the first systemic plasma concentration‐AEs study with the proposal of upper limit for the concentration‐dependent AEs of IM in GIST patients.

For IM, the concentration‐efficacy relationship has been investigated both in CML 7, 16, 17, 18, 19, 20, 21, 22, 23, 24, 25, 26 and GIST patients 8, 27, 28, 29, 30. There are some reports about the lower limit of IM as well. However, very few studies on the relationship of plasma concentration with toxicity have been reported, and no upper level limit has been formally defined, in particular in GIST patients. In 2012, Guilhot et al. investigated the plasma exposure of IM and its correlation with clinical response in patients with CML and found that there appeared to be an association between Cmin of IM (in the categorizing levels) and the frequency of myelosuppression with no upper limit of the therapeutic window determined yet 24. In 2014, Gotta V. et al. reported a trough concentration threshold of 926 ng/mL for the occurrence of overall AEs in CML 31, with ROC_AUC_‐values ranging between 0.54 and 0.64. This study reported that IM‐induced myelosuppression is plasma concentration‐dependent, not only in the categorizing exposure levels, but also over the whole range of trough concentrations. The estimated trough concentration threshold for myelosuppression was 1451.6 ng/mL with ROC_AUC_ (95% CI) of 0.693 (0.597–0.789) in patients with GIST, the potential of the discrimination threshold for myelosuppression in our study was in general better than those mentioned in Gotta V. et al. study. Certain studies have demonstrated that maintaining IM concentration above the limit of 1100 ng/mL seems to be crucial for achieving a good clinical response in patients with GIST 8. The therapeutic range of 1100–1451.6 ng/mL in patients with GIST needs to be verified further.

In this study, except for myelosuppression, which was found to be correlated with plasma concentration, other AEs, including edema, diarrhea, musculoskeletal complaints and fatigue were not found concentration‐dependent, either in the categorizing exposure levels or over the whole range of trough concentrations. Similar to our study, Larson RA. et al. reported that IM‐induced adverse reactions rates were similar among the IM quartile categories except for anemia, rash and myalgia 17. Furthermore, the lower frequency of rash and edema occurring among patients with the higher IM plasma concentrations (Q4) suggests that development of certain AEs may be less of a consequence of drug plasma concentrations, and more dependent on disease or disease stage. However, these results differed greatly with the results of Gotta V. et al. where significant associations with IM exposure were found for rash, fluid retention and gastro‐intestinal side effects. Therefore, the relationship between plasma concentration and AEs should be investigated further.

As mentioned previously, IM is metabolized predominantly by CYP3A4 to its active metabolite (CGP74588, NDI). Although NDI was reported to represent less than 20% of the circulating amount of parent drug at steady state, we found that it was significantly correlated with the incidence of myelosuppression (w/myelosuppression vs. w/o myelosuppression: 242.1 ng/mL vs. 206.3 ng/mL, *P *=* *0.006). Therefore, these results indicate that both IM and its major metabolite detection is necessary when considering TDM.

It is known that a good clinical response was associated with adequate plasma concentrations and the lower threshold for IM C_min_ has been established for improving outcomes. Our results showed that increased myelosuppression was correlated with high plasma concentrations and an upper level limit was proposed. These results strongly indicated that safety of IM could be improved for a subgroup of patients with GIST from dosage optimization using TDM.

## Conflict of Interest

The authors declare that they have no competing interests
